# Promotoras de Salud in a Father-Focused Nutrition and Physical Activity Program for Border Communities: Approaches and Lessons Learned from Collaboration

**DOI:** 10.3390/ijerph191811660

**Published:** 2022-09-16

**Authors:** Cassandra M. Johnson, Marlyn A. Allicock, Joseph R. Sharkey, M. Renée Umstattd Meyer, Luis Gómez, Tyler Prochnow, Chelsey Laviolette, Elva Beltrán, Luz M. Garza

**Affiliations:** 1 Nutrition and Foods Program, School of Family and Consumer Sciences, Texas State University, San Marcos, TX 78666, USA; 2 Department of Health Promotion and Behavioral Sciences, School of Public Health-Dallas Regional Campus, The University of Texas Health Science Center at Houston, Dallas, TX 75207, USA; 3 Department of Health Behavior, School of Public Health, Texas A&M University, College Station, TX 77843, USA; 4 Department of Public Health, Robbins College of Health and Human Sciences, Baylor University, Waco, TX 76706, USA

**Keywords:** colonias, promotoras, Latino fathers, family systems, health promotion, rural, community-engaged research

## Abstract

Promotoras de salud (promotoras) have been a valuable part of community research for Latino families, such as in the recruitment or delivery of health promotion programs. However, there has been limited discussion of how to integrate a promotora model into a father-focused program to support nutrition and physical activity within Latino families. This manuscript’s purpose is to describe how to engage and collaborate with promotoras in a father-focused, family-centered program for Latino families living in colonias near the U.S.–Mexico border. As part of a longstanding community–academic partnership, the authors outline approaches and lessons learned from collaboration with promotoras during the design (including formative work and training), implementation, and evaluation of a behavioral program—¡Haz Espacio para Papi! (HEPP, Make Room for Daddy!). Promotoras’ contributions supported the entire program, from design through evaluation. The team of all-female promotoras created a balance between the needs and preferences of the community and the goals and requirements of the research. While there is considerable time and human capital required for collaboration, the mutual benefits can make this work meaningful to all involved.

## 1. Introduction

In the Lower Rio Grande Valley of Texas, colonias are clusters of neighborhoods located on land previously used for agriculture along the U.S.–Mexico border; they are inhabited mostly by Latino/a individuals and families. Colonias are functionally rural communities in mostly unincorporated areas (technically extra-territorial jurisdictions), with limited access to affordable and nutritious foods [1,2]. Historically, colonias have been excluded from economic investments in infrastructure or amenities that support health and well-being [3,4], such as adequate housing with access to utilities (including electricity and water or sewers), paved roads, public transportation (including options for active transportation), parks, and recreational spaces and facilities. In Texas, Mexican-heritage adults and children living in border colonias experience persistently high poverty, high prevalence of food insecurity, suboptimal dietary intake, and diet-related chronic diseases [3,5,6]. Moreover, prior research has demonstrated barriers to physical activity within the community, including access to safe physical activity resources as well as environmental concerns such as heat and unleashed dogs [7,8,9]. Sharkey and colleagues have published several research studies describing the challenges of and opportunities for supporting nutrition and physical activity with Mexican-heritage families living in the colonias of south Texas [5,6,7,10]. Additional work has identified social and structural supports for physical activity present within the home and community [7,8,9,11,12]. Prior research has also documented the strengths of Latino families and communities for health promotion, such as collaboration with promotoras de salud (promotoras) to build on socio-cultural values related to family (or family values) and promote healthy behaviors [13,14,15].

As defined by previous research articles, promotoras are female community health workers (CHWs) who work in Latino and Hispanic communities [16,17]. They are trusted members of their community. Promotoras typically share common ethnicity, socio-cultural values, language, and life experiences and reside in the community they serve [16,17]. As part of research studies, promotoras can share knowledge with participants, provide emotional and instrumental support and encouragement to build skills, and increase self-confidence for skills or behaviors [13]. Because they are trusted and part of the community, promotoras can engage and interact with participants, supporting recruitment and retention. In addition, promotoras can act as a cultural and linguistic bridge between native Spanish-speaking participants and English-speaking researchers. Due to their educational and occupational training and experience, promotoras can also navigate different environments with ease [18].

In the state of Texas, the Department of State Health Services (DSHS) provides training and certification for promotoras via a promotor(a) or community health worker training and certification program [19], which enables certified promotoras to assist with research studies [16,17]. By building a strong rapport with those they serve, promotoras have a unique role in advancing health, a role other health professionals may not be able to provide. Promotoras can assist in conducting research respectful of community norms and traditions while still maintaining the rigor needed for academia. When promotoras and university-based researchers work together throughout a research study, they can influence the training materials, approaches, and instruments and allow the study to be more culturally relevant [16,17].

Given the opportunities and potential benefits of promotora engagement, promotora models have been incorporated into health promotion research on border communities [3,20]. While academic-based researchers can engage promotoras as research partners through the research process [16,17], there is less known about how to collaborate with promotoras and integrate their perspectives throughout the phases of a behavioral program to support nutrition and physical activity. This manuscript describes participatory collaboration with promotoras as research partners in designing, implementing, and evaluating a behavioral program—¡Haz Espacio para Papi! (HEPP, Make Room for Daddy!)—and lessons learned from the collaboration. The purpose of the current manuscript is to share practical insights from a promotora model and behavioral program with Latino families in border communities, such as notes from the field.

## 2. Integrated Approach to Collaboration

This section describes an integrated approach to collaboration with the promotoras and the lessons learned during program design, implementation, and evaluation.

### 2.1. Overview of the HEPP Program

A separate article in this Special Issue describes the rationale and design of the ¡Haz Espacio para Papi! (HEPP, Make Room for Daddy!) program [21]. The HEPP program was part of a large U.S. Department of Agriculture grant called Salud para Usted y Su Familia (SPUSF, Health for You and Your Family), which focused on health promotion for Mexican-heritage families in south Texas (USDA NIFA 2015-68001-23234). Briefly, eligible participants for the behavioral program were: parents (male and female) 21 years old or older and self-identifying as Mexican (participant, parent, or grandparent born in Mexico); parents who preferred to speak, read, and write in Spanish; cohabitating with partner or spouse and a child between 9 and 11 years at enrollment (or the start of the program); able to complete in-home measurement visits pre-and post-program and commit to full participation in the 6-week program; and parents who had lived in the colonia (or neighborhood cluster) for at least one year [21].

Importantly, the HEPP program is part of a longstanding and existing collaboration. Since 2006, academic-based researchers have engaged and collaborated with several community advisory boards (CABs), leaders of community organizations, a team of promotoras, and families living in colonias near the Texas–Mexico border, specifically in Hidalgo County, Texas [22]. Additional articles have described the outreach, education, and research activities in the study area [3,17,23].

**Figure 1:**
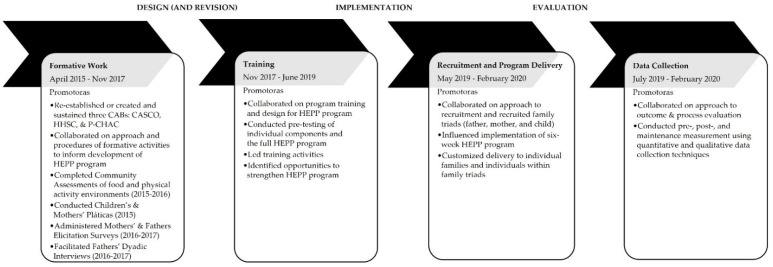
Timeline and promotora engagement throughout the design, implementation, and evaluation phases of the HEPP program. This figure shows how promotoras were engaged in different phases of the HEPP program. Due to the COVID-19 pandemic, the program stopped in late February of 2020. Pláticas were a series of group conversations. CAB: community advisory board. CASCO: Advisory Committee for Health and Community. HEPP: Haz Espacio para Papi (Make Room for Daddy). HHSC: Hand-in-Hand in San Carlos). P-CHAC: Progreso Community Health Advisory Council.

A team of academic and community researchers collaborated to design, implement, and evaluate the HEPP program for Mexican-heritage families in south Texas. Figure 1 presents key program activities between 2015 and 2020. The multisite research team included faculty members, graduate and undergraduate students, and promotoras de salud (promotoras), who were specially trained community health workers. The authors have previously published articles describing promotoras as partners in community-based research projects [16,17]. Overall, team members represented different racial and ethnic backgrounds and genders and included people who had lived or worked in rural communities. Several team members were bicultural and bilingual (English and Spanish language skills). In addition, team members had extensive experience with community engagement, outreach, evaluation, and research.

#### 2.1.1. The Team of Promotoras

The group of promotoras consisted of female community health workers who lived in the study area and self-identified as Latina or Hispanic. At the start of the SPUSF grant, there were three core (full-time) promotoras who had worked with the research team for several years. Their educational and occupational training and experiences varied but included teaching and health education experience, public health experience, and social work. The women ranged in age, from mid 20s through upper-40s. All three core promotoras were born in Mexico and were native Spanish speakers. Out of the three, one was fully bilingual (English and Spanish), another had professional working proficiency in English, and the third one had limited proficiency in English. Two of the three core promotoras were certified community health workers (CHWs). This team of three promotoras co-led the formative work. Two core promotoras co-led program training, implementation, and evaluation. These two promotoras were both bilingual.

#### 2.1.2. Hiring Process for Promotoras

Two core promotoras co-led the program activities related to design, implementation, and evaluation. These two promotoras were both bilingual. To increase staffing to meet the future demands of program implementation and evaluation, during the latter part of the design phase and throughout the implementation and evaluation phases, new team members who were either CHWs or had served as a CHW in the program joined the team by referral (word of mouth) or by applying to the program’s job postings. In total, this accounted for eight part-time team members.

The potential promotora candidates participated in interviews, where the program coordinator and current promotoras could assess their experience and skills, especially their interpersonal skills. The selection process consisted of an interview (to understand their lived and work experiences) and an evaluation (to assess their skills, e.g., communication and problem-solving) and lasted about 2.5 h. The interview focused on understanding their current and past experiences working with the community and with peers or colleagues. The evaluation focused on using objective methods to assess language skills (e.g., proficiency in English and Spanish), communication (e.g., delivering informal education to potential community members), and problem-solving skills.

### 2.2. Design—Formative Work and Training

#### 2.2.1. Design

Academic-based team members utilized published research findings, previous experience working with Latino families, formative work, and promotoras’ insights to create the HEPP program. The program was developed simultaneously in English and Spanish, with the support of a dedicated linguistics team for translation and transcription. At the beginning of the program design, the team followed an iterative process of development, review, and revision, where the promotoras and academic researchers worked together to create the program. They provided feedback on the program structure and curricula for nutrition and physical activity, including program activities and materials. As the program structure and curricula emerged, a cyclical process of review, training, and revision was used to modify the program components before pre-testing with a group of volunteer families. Formative activities that informed the HEPP program included activities with community advisory boards (CABs), community assessment, pláticas, household elicitation surveys, and dyadic interviews. The next section describes the approach and lessons learned from the formative work.

#### 2.2.2. Formative Work

Formative work to inform program design focused on understanding the priorities related to nutrition and physical activity (including needs and assets) of different family members (e.g., fathers, mothers, and children) and identifying valuable strategies for recruitment. Formative work began four years before program implementation in July of 2019 and included community engagement with three different community advisory boards [8]. Figure 1 shows activities from formative work. Although one CAB (Progreso Community Health Advisory Council (P-CHAC)) had been previously established with a prior project, the promotoras initiated the creation and maintenance of two additional CABs in 2016 (final meetings in 2020). P-CHAC hosted its first meeting in 2015. The other two CABs ((Advisory Committee for Health and Community (CASCO) in San Juan and Hand-in-Hand in San Carlos in San Carlos (HHSC)) were established and had their first meetings in 2016. Together, the CABs serve to advocate for the priorities and preferences of community leaders and members and provide feedback on the HEPP program.

At the same time, the team initiated a comprehensive community assessment between 2015 and 2016, which included extensive ground-truthed mapping of the community food environment and physical activity environment in study areas. Ground-truthing included area institutions, community food programs, community resources, programs, schools, street segments, and transportation. The promotoras led the formative work. They collected quantitative and qualitative data with Mexican-heritage fathers, mothers, and children living in the study area using the following data collection techniques: pláticas (defined as group discussions), household elicitation surveys (interviewer-administered surveys), and dyadic interviews (interviews between two participants). The initial formative work started in September of 2015 with children’s pláticas, and the final formative work activity finished in October 2017. The team of promotoras conducted all formative work activities in person and in Spanish and English between 2015 and 2017, and a linguistics team applied a two-stage process for transcription and translation.

Children’s Pláticas. As part of the formative work, the promotoras completed children’s pláticas, a panel series of three sequential group discussions with children who were 8 to 11 years old. The purpose of children’s pláticas was to understand the children’s perspectives related to preferences and family support for nutrition and physical activity. The same group of children completed all three discussions. Each discussion was organized around a different topic and incorporated visual activities. Session 1 focused on food-related activities and included “draw, write and tell” and “circles of closeness” activities. Session 2 covered physical activity, active play, and screen time with “map and tell” and “circles of closeness” activities. Session 3 included participant-driven photo elicitation (PDPE) [10], which is a process of training participants to take photographs of food-related activities and physical activity, and interviewers (the promotoras) using their photographs as visual prompts for eliciting discussion. In total, there were 72 sessions (24 total panel series), with 89 children completing all three sessions in their panel series of pláticas. Twelve groups were conducted in Spanish and 12 groups in English. The promotoras audio-recorded the pláticas (≈108 h) and created verbatim transcripts using a rigorous transcription and translation process.

Mothers’ Pláticas. The formative work included a similar panel series with mothers to understand their perspectives on nutrition and physical activities within their families Promotoras led mothers’ pláticas that met three times over four weeks and included the same three-session foci described in the children’s pláticas above. In total, there were 51 sessions (17 total panel series), with 91 mothers completing all three sessions of the panel series. Forty-eight sessions were conducted in Spanish and three in English. Approximately 89 h of audio recordings were transcribed verbatim and translated using the same process.

Household Elicitation Surveys with Mothers. Promotoras conducted household surveys with mothers living in the study area. Another article reports on the interviewer-administered surveys with mothers [24]. Promotoras walked neighborhoods or went “door to door” and identified potential respondents based on the eligibility criteria that we used in the HEPP program [21]. Data were collected from 334 mothers using open- and closed-ended questions. Questions included separate items for children and their spouses that were related to eating and dietary behaviors, physical activity behaviors, social media and internet access, family characteristics, and participation in nutrition assistance programs. In addition to recorded responses, the promotoras also documented their observations during the interviews. All data were entered into a searchable database.

Fathers’ Dyadic Interviews. The promotoras recruited fathers using multiple methods. Most, if not all, of the fathers were the partners or spouses of the mothers who participated in the mothers’ pláticas or mothers’ household elicitation surveys or were fathers to some of the children who participated in the children’s pláticas. Additional recruitment approaches included door-to-door canvassing (or block walking) or re-connecting with households that had previously participated in a research study and consented to be re-contacted for a future study. The fathers did not know each other prior to the interview.

The purpose of the dyadic interviews was to create a comfortable opportunity for fathers to discuss their perspectives, challenges, and desires related to food, activity, and health in their families with other fathers. Promotoras helped facilitate conversations between two fathers, which offered opportunities for the in-depth discussion of nutrition, physical activity, and family topics.

In total, 31 fathers completed 15 dyadic interviews, and 1 father completed a one-on-one interview. The dyadic interviews lasted from 1.25–3 h each; the length was determined by the participating fathers. Interviews were held in spaces considered to be neutral or comfortable to fathers, such as local community centers and churches. Interviews were held near their homes to address transportation needs and accommodate work commitments. All interviewers were in Spanish. The interviews were audio-recorded. Transcripts were created using the same transcription and translation process. A separate manuscript has reported initial findings from the fathers’ dyadic interviews [25].

Household Elicitation Surveys with Fathers. Importantly, insights from the fathers’ dyadic interviews motivated additional data collection—the household elicitation surveys of fathers. Before that point, formative work only included the perspectives of children and mothers. However, promotoras realized that they had learned a great deal from the fathers’ dyadic interviews and wanted to better understand fathers’ perspectives. Promotoras completed interviewer-administered face-to-face surveys with 300 fathers who were recruited from the study area [25]. They collected similar data to the mothers’ elicitation surveys (e.g., eating and dietary behaviors, family characteristics, etc.). A separate article reports on data from these surveys [25]. All data were handled in a similar manner to the mothers’ elicitation surveys.

#### 2.2.3. Lessons Learned from the Formative Work

There were three lessons learned from the formative work. First, the promotoras leveraged their knowledge and skills to develop customized recruitment strategies to center family values and prioritize relationships with children. Second, they identified ways to connect with mothers and fathers in different ways during recruitment. Third, the promotoras created additional opportunities to give back to the community by forming and sustaining new community advisory boards (CABs), which promoted trust and facilitated the academic–community collaboration. Promotoras identified potential barriers to connecting with community members or completing data collection activities and generated creative solutions. For example, during the formative data collection, the promotoras incorporated a “playroom in a bag” into the formative activities to make settings and activities more child-friendly. The promotoras also developed a “playroom in a bag” to re-create a welcoming environment in different settings. Components included lounge pillows, an interlocking foam puzzle floor mat, and pre-session games. Overall, the formative data collection activities allowed the promotoras to collect data, review materials, iteratively provide feedback to the research team, and then develop a customized recruitment strategy for connecting with individual families and specific family members (e.g., fathers, mothers, and children). Collaboration with promotoras during formative work helped ensure that the formative work activities would be well received by the community; this benefited both the research project (behavioral program) and the community.

**Lesson #1: Customized recruitment approach to fathers to center family values and prioritize relationships with children.** During recruitment for the formative activities, the promotoras intuitively built support for the program by working with fathers, their children, and their partners or spouses. They knew that fathers would be interested in the program if their children wanted them (the fathers) to participate. In early conversations with children, promotoras focused on chances to have fun and spend time with their fathers. One promotora said: “In Latino families, especially with fathers, children are the best agents of change”. The children seemed to encourage fathers to “step out of their comfort zones” and consider enrolling in the program. The recruitment efforts (for the formative activities) improved as promotoras obtained the “buy-in” of the rest of the family, especially the children.

Feedback from the promotoras about the importance of family values and developing relationships with their children also motivated decisions regarding program design. For example, the program included more children-focused and father–child activities to strengthen the bonds between fathers and children. At the same time, the program considered the reciprocal interactions between fathers and children, where fathers may have been more willing to try something new because their children felt excited and wanted them to participate. Promotoras also knew how important fathers were to their children and wanted to see the program explicitly show how the fathers could lead activities related to nutrition, activity, or health in their families. Their feedback informed the program design and decisions related to implementation.

**Lesson #2. Identified different ways to connect with mothers and fathers during recruitment.** Another observation by promotoras from the formative work related to misunderstandings about the fathers’ interest and motivation to engage in health promotion. During recruitment, promotoras reached out to fathers after first connecting with the mothers and children. Specifically, promotoras went door-to-door to talk to the mothers about the program’s value and presented the program to children as a fun way to spend time with their fathers. After this initial connection, promotoras followed-up with fathers by phone/text/in person to present the program and gauge their interest in participating.

While there have been prior efforts to engage fathers [26,27,28], there remains skepticism about fathers’ motivation and willingness to participate in more gendered activities related to health and food specifically. Traditionally, in Latino families, anything related to food and cooking inside the home is considered the domain of mothers. Conversations with mothers and fathers during the formative activities indicated that this skepticism (along with criticism and cynicism) negatively affected fathers’ intentions and behaviors. In previous research, fathers reported that the main reason for not participating in studies or programs was that they had not been asked [26,29,30]. Because the promotoras understood the importance of ensuring that the program engaged fathers in meaningful ways, formative activities focused on individual family members separately, such as conducting pláticas with mothers or dyadic interviews with fathers only. In addition, promotoras’ insights were central to the program structure, where the first two sessions focused on the family as a system, built the mothers’ support for the program, showed the value of the program to the fathers, and then shifted explicitly to a father focus in session three.

Promotoras guided the recruitment approach of fathers and ensured that the process was culturally appropriate and did not discourage or exclude potential families. The promotoras felt strongly about getting buy-in from the mother of a family before connecting with anyone else in the family. From the promotoras’ perspectives, the women/mothers are the gatekeepers to the family. The promotoras shared information about the program and their intentions, which allowed these women/mothers to learn of the value of their partners/spouses participating in the program. These conversations allowed the women/mothers to ask questions and share their intentions of communicating with their partners/spouses about the program. Without these conversations, promotoras were concerned with the potential for misunderstandings regarding why promotoras were reaching out to someone else’s partner/spouse. The promotoras communicated the importance of creating a welcoming space for fathers, so they could feel supported as they learned more about nutrition, physical activity, and health. During the formative activities, promotoras created two “pitches” (or “elevator speeches”), one for fathers and one for their spouses/wives. The fathers’ pitch highlighted why the fathers would want to participate in the program, considering the context of their culture and traditionally gendered norms. Their pitch (to the fathers) acknowledged the spouses/wives as gatekeepers within families, presented the program as an opportunity for fathers to spend time together with their families, and emphasized the fun activities related to food and physical activity.

In addition, communication between promotoras and participants emerged as a critical part of engagement for recruitment during the formative work and later during program implementation. During the formative work, participants commented on the promotoras’ communication with them (and the community in general) and the ways in which the promotoras’ interpersonal interactions aligned with the community’s values (e.g., respect, family). Because the promotoras were from the community and lived there, they were already navigating local culture and spoke in ways the community understood and trusted. This inherent knowledge and language allowed them to customize how they presented the formative work to the families and later generated insights for presenting the program to potential program participants.

**Lesson #3: Created additional opportunities to give back to the community through community advisory boards (CABs).** Finally, the promotoras further provided guidance and insight on community engagement through the CABs [8]. Previous literature has described how community engagement, specifically with CABs, can promote trust and enhance the relationships between academic- and community-based researchers [31]. The promotoras facilitated the creation and maintenance of three different CABs, which consisted of community members and leaders affiliated with local churches, county and town services, and other community-based organizations. Initially, the CABs focused most of their efforts on issues related to the SPUSF grant and a smaller proportion of their efforts on other community priorities. However, the promotoras supported the CABs in identifying their own priorities. Over time, the CABs allocated more of their time to their own priorities while also supporting the grant activities. The CABs were considered equal partners to the research team. The promotoras’ contributions to the CABs built trust and enhanced community relations.

Through their work with the CABs, the promotoras secured locations for research and outreach activities, including the program site at the Endowment Community Center. The promotoras also influenced the research activities by centering the community’s interests. For example, CAB members would share concerns, ideas, or feedback, and the promotoras would incorporate the community’s interest in their activities. Giving back to the community with resources and valuable activities was key for the promotoras. Another example was the promotoras’ approach to establishing new relationships with community members. With their focus on helping the community and understanding community values and culture, they went into new communities with donations and distributed donations to meet residents. Activities like this supported the grant’s activities but also went much further in bolstering community relationships.

#### 2.2.4. Additional Community Activities

While not part of the formative work to inform HEPP’s program design, the promotoras organized and hosted charlas, or friendly conversations, as part of community engagement and outreach (January 2017–July 2018) during the pre-design phase (Figure 1). The charlas provided health information to the community. Prior research has described the value of charlas when working with Hispanic and Latino communities [32,33]. The charlas focused on five different topics related to nutrition, physical activity, and mental health (e.g., stress, anxiety). The topics were: (1) healthy portions; (2) understanding dietary fats and their effects; (3) saving money and eating healthy; (4) healthy mind, healthy body; and (5) living without anxiety. The promotoras led charlas in community centers, churches, and residents’ homes and offered separate charlas to adults and children. In total, the promotoras held 63 charla sessions, with 731 adult participants (including individuals who attended more than one charla). The promotoras identified 39 male participants who attended the charlas. This kind of community engagement helped to build trust, identify potentially eligible program participants, and prime the community for future health promotion programs. This was an extensive ripple effect of the charlas.

#### 2.2.5. Training

In the HEPP, the promotoras delivered the program and prepared for their roles as group leaders/interventionists through intensive training. The training offered a valuable opportunity for the promotoras to collaborate. Prior research has emphasized the importance of equipping group leaders who deliver the program with the knowledge and skills to value fathers’ contributions as parents and co-parents and reduce biases related to mother-based child-rearing practices [34]. Given the gender discordance between female promotoras and male participants, training for the HEPP program focused on applying strengths-based approaches and aspects of motivational interviewing (MI) to create a non-judgmental and supportive experience for fathers. Critical considerations in the training were to ensure that promotoras were prepared for their role as group leaders and able to maintain knowledge and skills over time.

Most training activities occurred between January 2018 and June 2019, which was before the launch of the program in July 2019 (see Figure 1). However, there were additional training sessions for post-program and maintenance measurements between August 2019 and January 2020. Training activities included mini-presentations, games, discussions, demonstrations, role-playing, guided and self-study activities, observations with feedback, and booster training. In total, promotoras completed approximately 537 h of training for the HEPP program, not counting the time spent reviewing materials independently or completing training assignments at home. Thus, the estimate of total time is conservative. The total time spent in training was far greater than a previous program that applied a promotora model (e.g., HEPP: 537 h in training versus Aventuras para Niños: 22 h [35,36]). Supplementary Table S1 provides details on the training activities.

Almost all training activities were completed in-person and over multiple days (see Table S1). Some booster training sessions were delivered remotely via video conference apps (e.g., Webex or Zoom). Initially, academic-based researchers designed and led training activities for the core promotoras with principles of dialogue-based adult learning, such as building lessons with the 4 As: Anchor, Add, Apply, and Away [37] and with the promotoras’ preferences and needs in mind. The lead trainers for nutrition (C.J.), physical activity (T.P. and M.R.U.M.), and motivational interviewing (M.A.A.) had prior training experience. Parts of the nutrition and physical activity training included previously recorded videos to support the in-person training sessions. In addition, training activities included team-building events such as meals together or local sight-seeing activities, when possible, and often concluded with wrap-up games and prizes. As the training processes moved forward, the core promotoras helped develop and lead training for the promotora team (see Table S1). Promotoras received compensation for their time spent training (e.g., attending sessions, homework) and were reimbursed for travel to and from training sites [35,36].

Training topics covered foundational terms and concepts in nutrition and physical activity, the theoretical framework for the program, and a strengths-based approach to program delivery, which was informed by principles from MI, in addition to hands-on training to prepare promotoras to lead cooking lessons and physical activity segments and collect evaluation data for pre-, post-, and maintenance measures. Overall, the training attempted to increase their knowledge of nutrition, physical activity, and social-cultural influences on nutrition and physical activity behaviors; it increased their skills related to supporting behavior change, preparing food, and engaging in movement and exercise safely and efficiently. There were also training activities to prepare promotoras to deliver the mothers’ charlas, which were educational lessons with art and crafts activities. The mothers’ charlas occurred concurrently with the father-focused program sessions; however, the charlas happened in different rooms. A separate article provides detail on training activities for the physical activity parts of the program [38].

There were specific training sessions on food preparation for the cooking lessons and movement and exercise for the physical activity segments. Parts of the training emphasized techniques for ease of movement and safety to minimize injuries. Training included demonstrations led by the nutrition and physical activity leads and research staff and opportunities for the trainers to “walk through” the different lessons with the promotoras. All training provided ample time for discussion. The training made time for the promotoras to provide feedback and ask questions. The promotoras also participated in role-playing, where they functioned as the lesson leader and delivered different program components. Lastly, the training sessions focused on how to set up and use the devices for program evaluation. The program assessed primary outcomes with the Veggie Meter^®^ (Longevity Link, LLC, Salt Lake City, UT, 84108 USA) and ActiGraph GT9X accelerometers (ActiGraph, Pensacola, FL, 32502 USA) for nutrition and physical activity, respectively. Briefly, the Veggie Meter^®^ is a rapid, non-invasive device that measures skin carotenoids, which is a biomarker for dietary intake of fruits and vegetables. Accelerometers are a wearable technology that measures physical activity.

After several rounds of review, training, and (re)design, the promotoras completed several practice sessions of the HEPP Program with the research team (Table S1). Practice sessions occurred in the Endowment Center, which was the location of the actual program. This final round of training allowed the promotoras to practice delivering all the major lessons (e.g., interactive lessons, physical activity segments, cooking lessons, eating together with goal-setting) before pre-testing. Promotoras used leader’s guides and timers to ensure that sessions were delivered per protocol and within the allocated amount of time. During the practice sessions, academic researchers made observations about the delivery, providing feedback, and promotoras identified additional opportunities to refine the program.

Regarding the MI training, a consultant hosted an initial two-day training that focused on practical skills related to open questions, reflective listening, and summarization. The promotoras received initial two-day training, completed homework assignments, and participated in booster training (in August 2018 and February 2019). Pre- and post-tests, along with recorded practice conversations, were used to assess fidelity to motivational interviewing skills and identify areas for improvement.

#### 2.2.6. Lessons Learned from Training

There were three lessons learned. The training identified: (1) a need for an iterative process for program refinement; (2) the importance of customizing training to the promotoras; and (3) that the process of training provided an opportunity to extend intervention effects.

**Lesson #1. A need for an iterative process for program refinement.** During the training, it became clear that training offered opportunities to refine the program, and an additional step of training was added to the iterative process: draft, review, train, and revise. This emergent approach reflected the team’s commitment to CBPR and the principles of cultural humility [39]. While this kind of iterative process—design, review, and revise to incorporate feedback from the review—is common in community research [40,41], the HEPP training process expanded this approach to include training.

As the academic team members prepared for the initial training sessions, the promotoras identified important priorities. A key concept was to provide training that would enhance promotoras in their helping role rather than have them “mimic” research professionals and to do so in a way that was credible and efficient [42]. Many changes were initiated from conversations between promotoras and academic researchers before, during, and after training sessions. Table 1 presents examples of changes to the nutrition curriculum that were suggested or influenced by the promotoras (e.g., changes to recipes). Prochnow et al. have previously described examples of changes to the physical activity curriculum [38]. The next paragraphs outline changes made to the program in general or the approach to experiential nutrition education.

For example, the promotoras were concerned about affordability during the review of the recipes and cooking lessons. As a result, the nutrition lead (C.J.) obtained food prices from a popular local supermarket (H-E-B) and calculated the total cost for preparing the entire recipe and the per-serving cost. The promotoras approved the affordability, and the recipes were integrated into the program.

Another example was the final session (Session 6), where the promotoras believed it was important to celebrate the family’s journey in the program. From their perspectives, the families had committed considerable time and energy to the in-person and at-home activities and deserved to celebrate their accomplishments. While training, promotoras shared their idea to improve the final session. Their feedback led to creating a memorable final session, complete with a circle of sharing, presentation of special family stones, completion certificates with family photographs, and celebratory cake.

One more example was from the practice run-throughs. The promotoras realized that Session 5 required participants to have more nutrition knowledge and skills with food labels. Through discussion, they identified an opportunity to integrate additional nutrition education into the program so that participants would be ready for the nutrition-focused Session 5. Specifically, the promotoras believed that more experience with MyPlate and reading food labels (including nutrition facts) would boost engagement in a traditional game called “toma todo” with nutrition education game cards. As a result, the nutrition lead worked with the promotoras to develop “tasting recipe lessons” for all sessions. Each tasting lesson included an important topic from the promotoras’ perspectives and supplementary educational handouts. The tasting lessons happened before the start of the 2.5-h session and progressively targeted the nutrition knowledge and skills required for Session 5.

In addition, training the entire team, including the promotoras, in MI affected the program design in a substantial way. The promotoras wanted cues to apply MI skills in group sessions, and after the MI training, the leader’s guide and activities were revised. For example, the academic team members notated and provided examples of where open questions could be asked, phrases that could demonstrate reflective listening during discussion exercises, reminders about remaining non-judgmental, and how to assess a participant’s motivation about the behaviors of focus. Because the MI training included more than just the promotoras, the team identified opportunities to apply this person-centered approach to the program design and strengthened the program. For example, post-training, the leaders’ guide scripts were rewritten to provide more open questions and check-ins, and lesson activities were revised to highlight families’ unique goals. Activities were reviewed and modified to incorporate MI skills more explicitly, such as the use of confidence and motivation rulers as part of the goal-setting activities. The promotoras also wanted cues to apply MI skills in the delivery of group sessions. Thus, academic researchers revised the leader’s guide and activities to enhance supportive opportunities for exploring health behavior changes within families.

**Lesson #2. The importance of customizing training to the promotoras.** Training is a critical element of promotora-delivered programs because it prepares them to provide important health information and interventions to underserved communities with limited access to health programs. During training, the academic-based researchers realized that accommodating promotoras’ preferences and goals was critical to supporting them as group leaders/interventionists and data collectors. The academic researchers tailored the training to individual promotoras based on language skills, preferences for training intensity (e.g., number and duration of sessions), and their existing knowledge and skills related to the program. For example, at the beginning of the design phase, one promotora had basic knowledge and skills related to food and nutrition, but she had minimal experience with food preparation and cooking. She did not feel comfortable leading the cooking lessons and wanted more opportunities to increase her knowledge and develop skills. The lead nutrition trainer provided additional booster training sessions and videos related to cooking lessons. Further, videos of the lead physical activity trainers conducting lessons were made for promotoras to view at their leisure to reinforce techniques and cues [29].

Aside from individual preferences for training, the team of promotoras also had preferences as a group of learners. For instance, the promotoras preferred to review materials in advance of the training and have opportunities to discuss the rationale for the decisions, ask questions, and provide feedback prior to engaging in more formal training activities such as demos, role-playing, and discussions. These strategies, including the review of the materials ahead of time, enabled them to feel more confident and prepared for implementation. The promotoras explicitly described the importance of being well-prepared for the training and wanted a say in the focus of the training. While the promotoras were interested in building the knowledge and skills required for program implementation and evaluation, they had additional goals for training.

For example, the promotoras communicated that their goal for the training was to benefit their professional development. While the academic team members focused on increasing the knowledge and skills required for the program, the promotoras’ wanted training to increase their confidence in program delivery and prepare them to help their communities. For example, the program coordinator acted as a liaison to request additional time for studying, providing feedback, and scheduling enough time for reviewing program materials and practice sessions with each other before completing role-playing with the program team. Further, the promotoras viewed the training as valuable professional development that would benefit them after the program and the communities they serve. This was especially true for the MI training.

**Lesson #3: The process of training provided an opportunity to extend the intervention effects.** Through observations, check-ins during the training, and training debriefs, it was observed that the promotoras’ involvement in training affected their self-efficacy, beliefs, skills, and health. The promotoras commented on changes they noticed in their own behaviors and the behaviors of their family members. Previously, Sanchez-Hucles and Sanchez have described reciprocal empowerment as a “leadership style of reciprocal respect, equality, and personal authority that is characterized by concepts such as mutuality, compassion, collectivity, engagement, and a consensus to enhance oneself and others (p. 216)” [43]. Having a first-hand preview of the program and its impact through the training deepened the promotoras’ commitment and enthusiasm about the program. For example, one promotora commented that before the program, she did not eat vegetable-based salads very often because she did not like them. However, with the different options for salads in the program’s set of recipes, she changed her mind. Not only did she start eating more salads, but she also prepared them for her family. Another promotora was nervous regarding her general cooking skills. By participating in these training activities and the program itself, she gained confidence and felt ready to engage more in food preparation and to cook for her family at home. Similar observations were made for the physical activity training as the promotoras showed tremendous progress in their abilities to perform many functional movements and continue to assist family participants while engaging in the activities themselves. For example, one participating family shared that their child led a workout session at home using the PA lessons provided in the program. They shared that both parents and siblings participated in the physical activity and that the child took photographs.

### 2.3. Implementation

The HEPP program consisted of six weekly in-person sessions focused on family functioning, nutrition, and physical activity, along with at-home activities and check-ins. A separate article presents the details of the program [21]. Before the promotoras offered the program to participating families, the promotoras pre-tested individual components of the program, including measurements, and then delivered the full six-session program to two volunteer families. All participants were provided an incentive to compensate for their time (see Table S1 for the timeline of pre-testing activities). Recruitment for the program started in late May 2019.

The promotoras played a critical role in implementation because they were trained as group leaders/interventionists and were wholly responsible for delivering the program. Program implementation reflected the promotoras’ desire to provide families with a truly family-centered program. In-person and at-home activities focused on supporting a family systems-level change rather than on behavior changes for only individual family members, such as the child or one parent. Promotoras’ feedback influenced the decision to allocate half of the sessions to the fathers and children and create distinct opportunities for the co-parents (or couple) to strengthen their relationship. For example, the decision to ensure that the first two sessions (Weeks 1 and 2) focused on the family triad as a unit (father, mother, child) and to begin the first father–child session in Week 3 was based on formative work and promotoras’ insights. Additionally, the decision recognized that mothers, especially those of Mexican heritage, tended to serve as gatekeepers for their families. Thus, allowing the family to start the program and complete the first two weeks together was essential for building trust and rapport with each of them, especially the mother, to later have her support and encouragement to ensure that the father- and child-specific sessions, starting in Week 3, would be successful. As for the reasoning behind the co-parenting session in Week 4, promotoras worked with academic-based researchers to discuss how to make space for the parents to learn strategies for supporting each other as partners and co-parents. The promotoras believed that explicitly focusing on co-parenting would facilitate positive long-term effects on nutrition and physical activity for families.

### 2.4. Lessons Learned from Implementation

There were four lessons learned specifically from implementation: (1) The importance of making fathers feel included and supported in their families; (2) a need to consider the language skills of fathers and children; (3) the importance of designing sessions with time for promotoras to engage in their own ways; and (4) the value of coordinating resources to meet families’ needs. Given that the role of the promotoras as partners was entrenched and committed to the success of the program within their communities, they were able to point out potential issues, provide specific solutions, and ensure that the approach was compatible with the families’ needs without compromising the program’s integrity.

**Lesson #1: The importance of making fathers feel included and supported as leaders in their families.** The promotoras felt concerned that the program might undermine the fathers as leaders in their families. They wondered if a father-focused program might make some fathers feel “less than” mothers based on their potentially low prior knowledge of nutrition and physical activity, existing food and nutrition-related skills, or past actions to model health-promoting behaviors in their families. They identified potential unintended consequences of fathers feeling embarrassed, uncomfortable, or isolated and communicated their perspectives to the team. In response, the program engaged fathers separately from mothers in some sessions (Weeks 3, 5, and 6) and worked to showcase fathers’ unique abilities and contributions to their families throughout the program.

**Lesson #2: A need to consider the language skills of fathers and children.** The promotoras noticed that some fathers had challenges with literacy. Some fathers had no reading ability, and, generally, limited literacy was more common among fathers than mothers. The promotoras stressed the importance of reliance on graphical illustrations and pictures, with visual aids and verbal presentations, as part of effective communication. The delivery of the program was modified so that the promotoras had time to check-in with fathers during session activities. This attention to the fathers’ literacy extended from implementation to evaluation, and the surveys integrated graphics and pictures to present response options for survey items. Applying strengths-based principles facilitated the promotoras’ inclusion of fathers and the validation of fathers as leaders (co-parents) in their families rather than focusing on deficits or weaknesses. Additionally, during the pre-testing of a trivia-style game in Session 6, the promotoras noticed that children with less-developed reading skills in Spanish were not participating in the game, which was presented visually in Spanish. This observation mattered because the team had assumed that bilingual children had similar skills in reading in Spanish and English. Based on the promotoras’ observations and suggestions for accommodation, visual materials for all activities were presented in Spanish and English and supported with verbal instructions in both languages.

**Lesson #3: The importance of designing sessions with time for promotoras to engage in their own ways.** Making time for promotoras to engage with the fathers was intentional. A key aspect of the implementation was ensuring that the weekly sessions had enough time for promotoras to tailor engagement to specific families and individual family members (e.g., walk around the room, personally check-in on all the family members, and offer support). In addition, the promotoras wanted to motivate the families to make changes related to nutrition and physical activity, which could also strengthen their families. However, it is important to say that these decisions would not have been made without explicitly recognizing the strengths of the promotora model and the individual strengths of the promotoras on the team. In the authors’ opinions, collaborating with the promotoras in the delivery of the program in advance offered unique advantages that would not have been available with group leaders from outside the community. However, making time for the promotoras to connect more one-on-one with families meant that they not only built rapport but learned of other needs the families may have had that could affect program retention. As such, promotoras created efficient procedures so they could spend more time communicating, engaging, and supporting families through a six-week program. For example, the promotoras reviewed processes for setting up, delivering, and breaking down/cleaning up post-program and made changes to streamline the process. In addition, the promotoras suggested that rather than shopping for the ingredients and other supplies themselves, the program place orders for curbside pickup. As another example, promotoras were able to reduce set-up time by developing an agreement with the community site (the Endowment Center) to store program materials on-site and requesting an additional storage room near the Endowment Center.

**Lesson #4: The value of coordinating resources to meet families’ needs.** Throughout implementation, promotoras were keen on pointing out potential barriers that would hinder family engagement and sought logistical support from the program coordinator and research team to resolve the issue. Their contributions emphasized the importance of coordinating resources to make it easier for families to participate. For example, the promotoras learned that some families were having issues attending the in-person sessions. Due to family or work demands or transportation challenges, some families, who attended the afternoon session, needed to make last-minute changes in their schedules to attend a morning session instead. The community center space was large enough to allow promotoras to accommodate more than the five or six families expected in each session. As another example, some families had challenges with transportation and could not drive their vehicles to the community center for a session. Given the lack of public transportation and limited access to rides (private transportation), families would have missed the session. The promotoras wanted the families to engage fully with the program, and, as needed, they coordinated and provided transportation to sessions. Another example is childcare. The program provided on-site childcare and activities for children of all ages, and children had access to an outdoor playground and recreation area behind the community center building. However, some children, who were the siblings of the participating child in the program, did not want to be separated from their parents or participate in the children’s activities. To keep families together and to remove barriers to engagement during sessions, additional staff would care for other children or guide them in arts and crafts activities or games while their parents and sibling engaged in program activities (in the same space). These adjustments promoted retention over the six-week program.

The promotoras, as group leaders and interventionists, were invested in their communities, wanted to help the families in their community thrive, and actively solved problems to encourage and enhance program engagement. Therefore, additional duties such as making phone calls to confirm attendance and arranging rides to or from the community center, rearranging the physical space for the different program activities (e.g., cooking lessons, physical activity breaks, etc.), preparing additional tasting recipes and ingredients for the main recipe for the additional families, and driving to people’s homes to pick up families and bring them to the community center were relegated to additional paid staff and volunteers. In this way, the promotoras focused on maintaining relationships with families and delivering a valuable program. Again, there are benefits to applying strengths-based and community-engaged approaches to program implementation in collaboration with the promotoras. The promotoras, as group leaders and interventionists, were invested in their communities, wanted to help the families in their community thrive, and actively solved problems to encourage and enhance program engagement.

### 2.5. Evaluation

As program partners, the promotoras helped guide the program evaluation. The program included pre-test, post-test, and short-term maintenance evaluations, completed three to four months post-program. The promotoras were involved in discussions related to both outcome and process evaluations. A separate article describes the outcome and process evaluations in more detail [21].

Briefly, the HEPP program utilized various techniques to collect quantitative and qualitative data for program evaluation. Primary outcomes included the dietary intake of fruits and vegetables (measured with the Veggie Meter^®^). Secondary outcomes included low and moderate physical activity (measured with accelerometers), family functioning (measured with FACES-IV [44,45]), and psychosocial factors related to eating fruits and vegetables. While the academic researchers selected the evaluation instruments and time periods, per grant protocol, promotoras provided feedback on the measures, procedures, and practical considerations related to data collection. The promotoras enhanced the evaluation process and ensured that it was culturally appropriate.

### 2.6. Lessons Learned from Evaluation

There were three primary lessons learned from the evaluations: (1) Minimize the respondent burden required for evaluation; (2) maximize one-on-one time with individual family members for mutual benefit; and (3) incorporate the promotoras’ perspectives on evaluation to value the family’s (qualitative) experiences of the program.

**Lesson #1: Minimize the respondent burden required for evaluation.** First, the promotoras advocated for paring back the data collection with surveys or other nutrition assessments to minimize the respondent burden. Longer scales, such as the Family Adaptability and Cohesion Evaluation Scale (FACES-IV) to assess family functioning [44,45], required more “convincing” to address initial resistance. On the other hand, the promotoras expressed more support for the Veggie Meter^®^ (Longevity Link, LLC, Salt Lake City, UT 84108, USA) because the device enables the rapid, non-invasive assessment of a biomarker for dietary intake. From a research perspective, the use of objective measures to assess outcomes, such as the Veggie Meter^®^, is recommended for reducing sources of measurement error. However, eliminating longer scales such as the FACES-IV (with 62 items) from the evaluation was a trade-off. Overall, the promotoras appreciated selecting measures or assessments that were convenient (e.g., easy to set up or complete) and fast (e.g., require little time).

**Lesson #2: Maximize one-on-one time with individual family members for mutual benefit.** During the evaluation visits, the promotoras wanted to have dedicated time with each individual family member to make the person more comfortable and collect evaluation data, which meant more coordination in scheduling the in-person home visits. The team modified the evaluation procedures so the three-member promotora team could complete in-person home visits and collect data separately for fathers, mothers, and children. This process allowed each promotora to focus on one family member at a time. From a research perspective, the one-on-one interactions also helped provide more support for family members to freely share about the program without the influence of other family members. Potential benefits of the one-on-one evaluation visits are potentially enhanced trust between the research team and the community and more honest responses to questions (less social desirability).

**Lesson #3: Incorporate the promotoras’ perspective on evaluation to value the family’s (qualitative) experiences of the program**. The promotoras believed that evaluation should prioritize interviews with individual family members (e.g., fathers, mothers, children) to capture the family’s experiences of specifically how the program has affected family functioning or dynamics. While changes in nutrition and physical activity outcomes (e.g., dietary intake of fruits and vegetables, low and moderate physical activity) were important, understanding the impact on the family experience was valuable to the promotoras. They believed that stronger family functioning could translate to better outcomes. This meant that the academic researchers included fewer quantitative assessments to assess outcomes and focused the evaluation instead on capturing families’ qualitative experiences with interviews. For example, most of the interview guide questions asked about family functioning or family dynamics (e.g., how the program has affected relationships between family members) rather than on individual-level behavior changes. While completing the interviews (as part of the evaluation), promotoras commented that fathers expressed their gratitude for being included and considering their perspectives, while mothers were grateful for the fathers’ opportunities to engage in the program and support mothers at home. Both fathers and mothers voiced how participation enabled them to consider things they had not before and helped them become more aware of ways to be a more supportive parent and partner. Participating children shared how they were able to spend time with their fathers and learn more about them. Research findings from the evaluation are forthcoming and will be reported in a separate article.

## 3. Discussion

As far as the authors are aware, HEPP is the first behavioral program that has explicitly focused on Latino fathers and families living in U.S.–Mexico border communities. To date, there have been two behavioral programs identified in the literature that have focused on Latino fathers and supported health-promoting nutrition, eating, or physical activity behaviors. The first is Padres Preparados, Jóvenes Saludables, an obesity prevention program for Latino fathers and early adolescents [46,47]. The second is Papás Saludables, Niños Saludables (PSNS, Healthy Dads, Healthy Kids), with Latino fathers and children [48,49]. PSNS is a cultural adaptation of the Healthy Dads, Healthy Kids (HDHK) program [50], which was initially developed by Morgan et al. for white fathers and their children who were living in Australia [51,52]. While there is limited evidence on best practices for how to engage with Latino fathers or how to support them in behavior change related to nutrition and physical activity [13,53], there is also limited discussion of how to integrate a promotora model into a father-focused behavioral program.

The team of promotoras was engaged throughout the program design, implementation, and evaluation. Apart from the process evaluation data, the program collected minimal data related to the promotoras’ engagement. However, because the promotoras and academic-based researchers worked together to design, implement, and evaluate the program, their contributions to the program were clear. There were many opportunities to review their knowledge and skills (or evaluate their activities), provide feedback, and support them as group leaders and data collectors. The process evaluation included frequent debriefings, team meetings, and opportunities to address emergent challenges. Additionally, positive experiences were evident during group sessions, check-in calls, and follow-up interviews. Participants felt especially impressed by the welcoming environment created by the promotoras. In addition, program participants regularly commented on how attentive, positive, and friendly the promotoras were during the program, which participants had not always experienced in other programs. In many cases, family participants started inviting promotoras to family gatherings and social events.

Considerable resources are required to do this type of work [54], which is grounded in the theories and best practices of community-engaged research, of engaging with Latino fathers directly and supporting health promotion in more remote communities such as border colonias. First, there is the human capital required to design the program, including formative work, training, translation services, and the additional cost of materials and personnel such as trainers, group leaders (or interventionists), and support staff required to pre-test and deliver this nutrition program. The intensive training was a limitation and would need to be addressed prior to future implementation. For example, during the 6-week program, there were 24 recipes prepared for each group of 10–12 families (at least three individuals per family). HEPP required equipment, supplies, including food purchased at retail prices, materials for the group sessions, and prizes for raffle drawings. Second, a program coordinator was key to managing logistics and facilitating communication between the academic-based researchers and promotoras, among other responsibilities. Third, the program would not have been possible without the ongoing support of community leaders, in particular, the director of the community center that was the site of all group sessions. While no fee was associated with the reservation or use of the community center’s meeting space for the group sessions, hosting the HEPP program displaced other community events that may have also benefited the community.

This collaboration has generated three overarching lessons learned for future community-engaged research. First, the partnership with promotoras is especially important as the promotoras presented solutions and developed strategies based on their lived experiences. Research teams may not have had the same experiences and, without the promotoras, may have missed opportunities to develop a meaningful program. Promotoras’ contributions supported the entire program, from design through evaluation. Second, the promotoras created a balance between the needs and preferences of the community and the goals and requirements of research. Specifically, this kind of collaboration provided opportunities for promotoras to offer their insights into the best approaches to meet the needs of the community while also maintaining scientific rigor. Third, the HEPP program demonstrated a well-trained team of female promotoras who understood the community, used strategic approaches in the formative work, and were able to co-create a program that engaged men successfully.

Researchers considering how to move forward with community-engaged research would benefit from building trust and relationships with community organizations, leaders, and families in the study area well in advance of the planned pre-testing and recruitment for the actual program rather than the typically allotted one to two years before a program begins. Our program benefited from and expanded the community partnerships developed over more than 10 years of research and outreach in the Lower Rio Grande Valley.

Additional time (for formative work, program design, and training) is critical to present and discuss ideas with promotoras and create opportunities for their insights to be integrated into the work. Because of the promotoras’ experience and perspectives, they identified solutions and implemented strategies that were unavailable to the researchers and, thus, made valuable contributions to the research project. Promotoras provided valuable insights into the appropriateness of research due to their knowledge and experience, which allowed researchers to develop and adapt the approaches and methods to benefit the community and the research study. The promotoras served as liaisons between the research team and community members throughout the grant and program process. This two-way communication ensured that the research project gained what was needed and was well received by the community, and the project and the community both benefited. With their knowledge and experience, promotoras ensured that the families and community benefited from the program.

Future research projects would benefit from early engagement and shared decision-making with promotoras. Without engagement and collaboration with the community members, including promotoras, there are missed opportunities not only to design an effective program but also to recruit and engage with potential participants and their families. In addition, there are risks of doing harm when programs are developed and implemented in ways that are misaligned with a community’s values, needs, and resources.

A community-engaged process that integrates a promotora model is essential for designing more effective behavioral programs. This team of promotoras guided design, implementation, and evaluation strategies that translated into a culturally meaningful program with high participant engagement. With their knowledge, skills, and commitment to their community, the promotoras implemented positive health behaviors at multiple levels—personal (self), family, and community. The promotoras maintained their dual focus on the goals of the community and research. Through this research collaboration, they fostered mutuality, where the promotoras could enhance their own skills and support their community in a meaningful way [55,56,57]. This type of community-engaged research requires a necessary paradigm shift that allows researchers to mitigate previous insensitivities and hurt while promoting a more equitable approach, which supports improved health equity for communities involved in and assisting in research [58].

## 4. Conclusions

Collaboration with promotoras was crucial to supporting behavior change at the individual and family levels, and the promotoras’ contributions to the CABs may have also promoted positive changes within the community. Thus, promotoras are essential partners in community-based research. The approaches and lessons learned that are described here demonstrated the mutual benefits of promotora engagement, from initial design through evaluation. Existing and emergent evidence from similar programs, in combination with the evidence from HEPP, can help identify opportunities for promotora collaboration as part of future health promotion efforts. Lastly, with limited evidence from father-focused programs designed specifically for Latino fathers, it is important to discuss what it means to design and implement a father-focused program with Mexican-heritage fathers living in U.S.–Mexico border communities. Community-engaged programs require sustained investment in the community and collaboration with community members, including promotoras, leaders, and the residents.

## Figures and Tables

**Table 1:** Selected examples of content changes to nutrition curriculum made in collaboration with the promotoras.

Program Change	Weekly Session	Rationale	Response
Used only limes (versus lemons).	All Weeks	Lemons had been originally offered as a variation for acid (in addition to limes) and were included in early drafts of recipes. The estimated price per ounce of juice was similar. Limes are smaller and less costly. However, because lemons are larger and yield more juice than limes, the price was similar. Originally, lemons were selected to increase the variety of ingredients. However, the promotoras insisted that limes were the preferred alternative. They believed that limes had important cultural significance to the families.	All recipes only used limes. No recipes used lemons.
Added meat filling to the tacos (versus vegetarian tacos).	Week 2	The promotoras wanted the second recipe in the program to make the fathers want to return for Week 3. Originally, the tacos were vegetarian and did not include meat. However, the promotoras were concerned about missing an opportunity to prioritize fathers’ preferences by including a dish with meat.	Modified recipe to include rotisserie chicken in the tacos.
Replaced tuna with chicken for the tostadas.	Week 3	Although a promotora had originally shared a recipe for a tostada with tuna (from another community event) and believed that the families would like it, the promotoras noticed that some families did not have positive responses to the dish during the implementation phase. The promotoras checked in with families and suggested chicken as an alternative.	Modified recipe to use canned chicken instead of canned tuna.
Replaced guava with mango in the vinaigrette.	Week 4	Guavas are a traditional fruit that can be included as another fruit (in addition to mango) in recipes. Promotoras liked the original recipe with a guava-based vinaigrette, but during training, there were challenges finding ripe guavas. In addition, working with guavas can be challenging for people, depending on their skills or experiences. The promotoras were worried that some people would not feel comfortable working with guavas in a new way (a guava-based vinaigrette) and wanted a different twist on a vinaigrette. The original recipe added guava as a twist to a lime-based vinaigrette.	Modified the main recipe to use the leftover mango (from the salad) to prepare the vinaigrette. No recipes used guava.
Maximized opportunity for children to have fun making and eating the recipes with edible sculptures.	Weeks 5 and 6	Based on the promotora’s experience, young children preferred playful recipes. She asked the team to include an edible sculpture as the main recipe for at least one session.	Designed two main recipes for the children: (1) Week 5: Vegetarian pinwheel, which was made from a filled tortilla and sliced cross-sectionally to reveal brightly colored fillings. (2) Week 6: Tuna “boats” complete with an edible mast and sail, which were made from a hollowed cucumber and filled with tuna salad.

This table presents some of the changes to the nutrition curriculum in collaboration with the promotoras. Most changes were made during the design phase, specifically during the review, training, and revision phase of the program and before the pre-testing of the program with participants. Separate manuscripts provide details on the nutrition curriculum and the recipes in the program [21] and changes to the physical activity curriculum [38].

## Data Availability

Not applicable.

## Supplementary Materials

The following supporting information can be downloaded at: https://www.mdpi.com/article/10.3390/ijerph191811660/s1, Table S1: Detailed training activities for the promotoras.
