# Supplementary material for: Promotoras de Salud in a Father-Focused Nutrition and Physical Activity Program for Border Communities: Approaches and Lessons Learned from Collaboration
Source: Int J Environ Res Public Health. 2022 Sep 16;19(18):11660. doi: 10.3390/ijerph191811660 (PMC9517298; doi:10.3390/ijerph191811660)
Supplement: Supplementary file 1 [file ijerph-19-11660-s001.zip › ijerph-1871680-supplementary.pdf]

Supplementary Table S1: Detailed training activities for the promotoras

Training Activity	Date	Duration	Mode
Physical Activity - Accelerometry (fathers)	Between 11/2017 and 01/2018	3 days (3 full work days, between 11/2017 and 01/2018)	In person
Physical Activity - Accelerometry (children)	Between 11/2017 and 01/2018	1 day	In person
Physical Activity - Accelerometry (Mothers)	Between 02/2018 and 04/2018	1 day	In person
Varied training activities (program goals and description of sessions)	04/24/2018 through 04/26/2018	3 days (3 full work days: 04/24, 04/25, and 04/26/2018)	In person
Family Functioning Survey Booster	04/27/2018	1.5 hour	Virtual
Physical Activity – Accelerometry (for non-removable accelerometers)	05/2018	2 hours	In person
Varied training activities (sessions 1-3)	05/23/2018 through 05/25/2018	3 days	In person
Motivational Interviewing (MI)	06/15/2018 through 06/16/2018	2 days	In person
Varied training activities for program, including new MI additions (sessions 1 - 3)	06/27/18 through 06/29/18	3 days	In person
Varied training activities for program, including new MI additions (sessions 4-6)	07/09/18 through 07/13/18	4 days	In person
Nutrition - Welcome recipes	Between 05/01/2018 and 07/31/2018	2 days	Virtual

Creation and review of videos focused on nutrition skills with recipe preparation, MI skills, and leading physical activity lessons	Between 05/01/2018 and 07/31/2018	NR	In person and virtual
Physical Activity – Accelerometry for new team members	08/2018	1 day	In person
MI Booster	08/09/2018	2 hours	Virtual
Nutrition - Shopping, food preparation, and cooking	08/13/2018 through 08/15/2018	3 days	In person
Nutrition - Survey (pre-program)	08/2018	2 days	Virtual
Nutrition - Anthropometry Booster	08/2018	1.5 hours	Virtual
Nutrition – Welcome recipes (with videos and promotora-led discussion)	08/2018	4 hours	Virtual and in-person
Varied training activities with focus on Nutrition - Shopping, making welcome recipes, delivering cooking lessons. Also, included practice, troubleshooting, and role-playing with interactive lessons	09/03/2018 through 09/07/2018	5 days	In person
Logistics, pre-program measurement, anthropometry booster training, role-playing with sessions, and completing full-session run throughs with debriefs	09/26/2018 through 09/29/2018	4 days	In person

Nutrition - Welcome recipes (practice, troubleshooting, and discussion)	10/2018	3 days	In person
Reviewing and role-playing with pre-program surveys for Family Functioning, Nutrition, and Physical Activity	10/2018	2 days	In person
Individualized training to review sessions one-by-one, practice delivering all components, and gain feedback (completed over three-week period)	11/2018	NR	In person
Varied training activities (including reviewing Nutrition Survey, Accelerometry, Anthropometry, and practicing with recipes)	11/2018	1 day	In person
Nutrition - Anthropometry. Pre-testing. Practice measuring height and weight with 30 individuals (10 fathers, mothers, and children). Included reviewing procedures, troubleshooting, and discussion	11/2018	NR	In person
Varied training activities (including shopping for materials and foods, practicing welcome recipes, full session run	12/10/2018 through-12/14/201)	5 days	In person

throughs, with focus on role playing, trouble shooting, and booster training for Veggie Meter, anthropometry, and accelerometry)			
Pre-program measurement, including role playing, troubleshooting, and discussion	01/24/19 through 01/26/19	3 days	In person
Booster training - Reviewing and practicing all sessions with feedback (completed over a 10-day period)	01/2019	NR	In person
Pre-testing sessions. Practice run-throughs of full sessions of six-week program for pre-tests (completed on six different days)	02/06/2019 through 03/22/2019	NR	In person
MI Booster	02/22/2019 through 02/23/2019	2 days	In person
Review, troubleshooting, and discussion of pre-program surveys for Family Functioning and Nutrition	03/18/2019 through 03/19/2019)	2 days	In person
Debrief interviews with participating families from pre-testing or practice run-throughs	03/28/2019 though 04/12/2019	NR	In person
Pre-testing sessions. Review and practice of pre-program measures,	03/2019	NR	In person

including role-playing with volunteers, troubleshooting, and discussion			
Nutrition - Veggie Meter; and Physical Activity – Accelerometry (data management procedures)	04/03/2019	3 hours	In person
Nutrition - Learn2Serve Food Handlers Training	04/2019	2 hours	Virtual
Pre-program measurement booster	04/24/2019	1 day	In person
Logistics (scheduling with program calendar)	05/2019	1 day	In person
Recruitment	05/2019	1 day	In person
Varied mini-trainings for additional team members	between 05/06/2019 and 06/21/2019	2 hours to 1 day per training	In person
Pre-program booster	06/05/2019 through 06/07/2019	3 days	In person
Pre-program booster	06/27/2019 through 06/28/2019	2 days	In person
Program starts	07/2019		
Measurement booster training	08/2019	4 hours	In person
Mini-training for new team members	Between 08/05/2019 and 10/22/2019	2 hours to 1 day per training	In person
Nutrition - Texas Food Handlers Course		2 hours	Virtual
Maintenance measurement	11/21/2019	5 hours	Virtual
Maintenance measurement booster	12/10/2019 and 12/11/2019	4 hours	In person

Call log training and booster training	01/16/2020 and 01/30/2020	1.75 hours	In person
Program ends	02/2020		
	Total time in training	536.75 hours	

The program coordinator (LG) provided records for activities and training sessions. The lead author (CMJ) extracted details from coordinator's documentation. When exact dates were not available, a date range was given. Training activities were to prepare the promotoras for their roles in the HEPP Program, and prioritized knowledge and skills related to the entire program, including theoretical foundation, nutrition and physical activity knowledge, and program outcomes, program structure and curricula, program delivery and evaluation. Generally, the academic-based researchers developed and led training, in collaboration with the core promotoras. Over time, the core promotoras led training for the other promotoras on the team. Total time did not include any activities where duration was not reported (NR). Calculation estimated 8-hour days, but many training days lasted beyond 8 hours. Thus, the estimate of total time is conservative.
